# Supplementary material for: Cooperative assembly of p97 complexes involved in replication termination
Source: Nat Commun. 2022 Nov 3;13:6591. doi: 10.1038/s41467-022-34210-y (PMC9633789; doi:10.1038/s41467-022-34210-y)
Supplement: Supplementary file 1 — Supplementary Information [file 41467_2022_34210_MOESM1_ESM.pdf]

Supplementary Information

Cooperative assembly of p97 complexes involved in replication termination

Olga V. Kochenova[1,2], Sirisha Mukkavalli[3], Malavika Raman[3], and Johannes C. Walter[1,2]

[1]Department of Biological Chemistry and Molecular Pharmacology, Harvard Medical School, Blavatnik Institute, Boston, MA 02115, USA

[2]Howard Hughes Medical Institute

[3]Department of Developmental Molecular and Chemical Biology, Tufts University School of Medicine, Boston, MA 02111, USA

Correspondence: johannes_walter@hms.harvard.edu

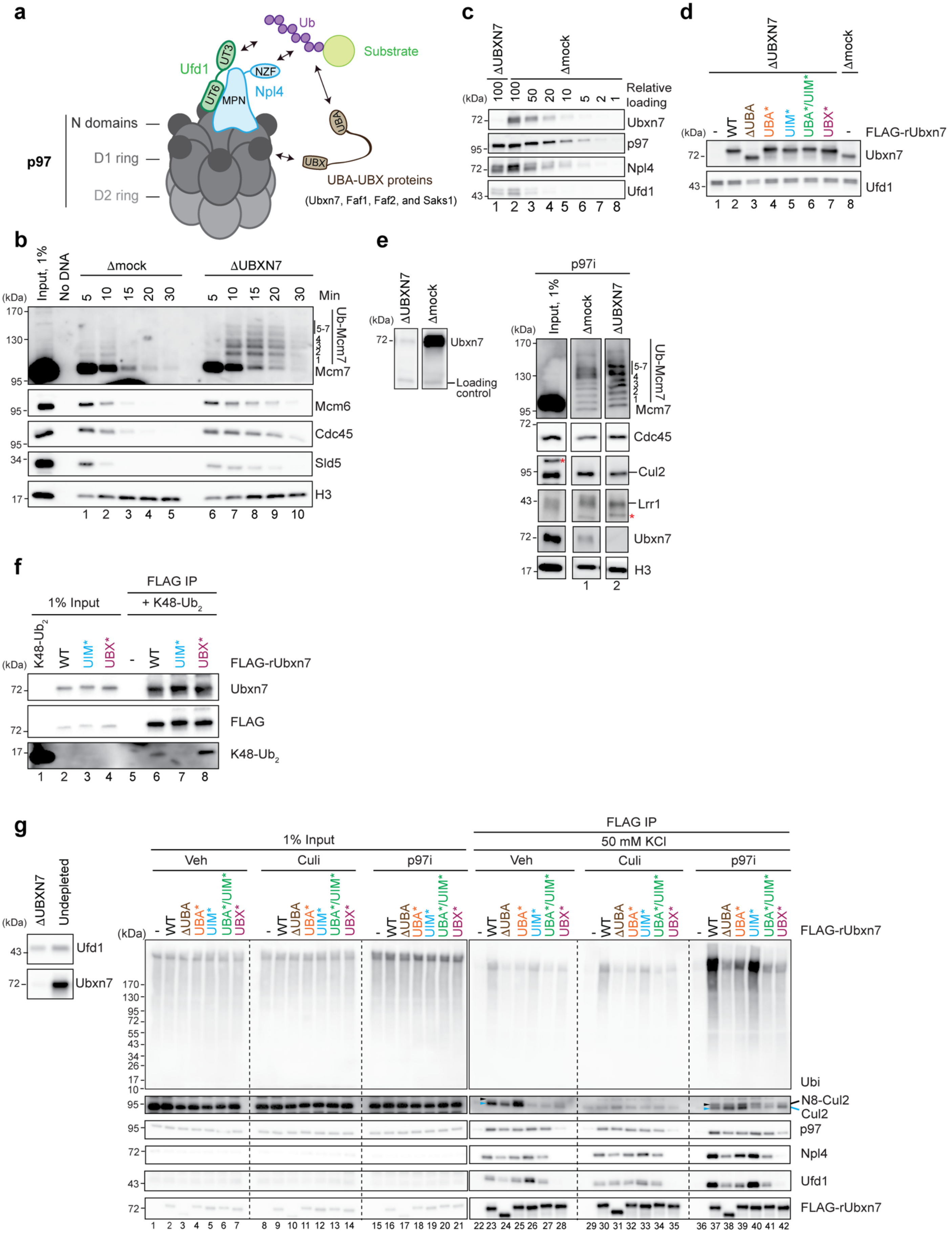

a
Ub
Substrate
Ufd1
UT3
UT6
NZF
Npl4
MPN
N domains
p97
D1 ring
D2 ring
UBA
UBX
UBA-UBX proteins
(Ubxn7, Faf1, Faf2, and Saks1)
b
Input, 1%
No DNA
Δmock
ΔUBXN7
Min
(kDa)
Ub-Mcm7
5-7
4
3
2
1
Mcm7
Mcm6
Cdc45
Sld5
H3
c
ΔUBXN7
Δmock
Relative loading
Ubxn7
p97
Npl4
Ufd1
d
ΔUBXN7
Δmock
WT
ΔUBA
UBA*
UIM*
UBA*/UIM*
UBX*
FLAG-rUbxn7
Ubxn7
Ufd1
e
ΔUBXN7
Δmock
Ubxn7
Loading control
p97i
Input, 1%
Ub-Mcm7
Mcm7
Cdc45
Cul2
Lrr1
Ubxn7
H3
f
1% Input
FLAG IP
+ K48-Ub2
K48-Ub2
WT
UIM*
UBX*
FLAG-rUbxn7
Ubxn7
FLAG
K48-Ub2
g
ΔUBXN7
Undepleted
Ufd1
Ubxn7
1% Input
FLAG IP
50 mM KCl
Veh
Culi
p97i
WT
ΔUBA
UBA*
UIM*
UBA*/UIM*
UBX*
FLAG-rUbxn7
(kDa)
Ubi
N8-Cul2
Cul2
p97
Npl4
Ufd1
FLAG-rUbxn7

Supplementary Fig. 1: Characterization of CMG unloading in Ubxn7-depleted extracts and the effect of Ubxn7 mutants. (a) Schematic showing the domain composition of p97, Ufd1-Npl4, and UBA-UBX proteins. Double arrows indicate known protein-protein interactions. D1 and D2 rings, ATPase domains D1 and D2, respectively; MPN, Mpr1, Pad1 N-terminal domain; NZF, NPL4 Zinc Finger domain; UT3, Ufd1 truncation 3 domain; UT6, Ufd1 truncation 6 domain; UBX, ubiquitin regulatory X domain; UBA, ubiquitin-associated domain. **(b)** Time course of CMG unloading in mock- and Ubxn7-depleted extracts. Plasmids were isolated at the indicated time points and immunoblotted with the indicated antibodies. Ub-Mcm7, ubiquitylated Mcm7. **(c)** Western blot analysis of mock and Ubxn7 depletions. Related to Supplementary Fig. 1b. **(d)** Western blot analysis of mock and Ubxn7 depletions and recombinant Ubxn7 (FLAG-rUbxn7) proteins add-back. Abbreviations as in Fig. 1a. Related to Fig. 1a. **(e)** Left, western blot analysis of mock and Ubxn7 depletions. Right, analysis of chromatin-bound proteins in mock- and Ubxn7-depleted extracts in the presence of p97i. Plasmids were isolated 45 minutes after replication initiation and immunoblotted with the indicated antibodies. The images are all part of the same Western blot, which was cropped to remove irrelevant information between lanes "Input, 1%" ,1, and 2. For Lrr1, the contrast of the whole image was adjusted using ImageJ to enhance visibility of the bands. Red asterisk, non-specific band. **(f)** Analysis of co-immunoprecipitates of wild-type (WT), I46A/L65A/L73A (UBA*), and P486G (UBX*) mutant variants of FLAG-rUbxn7 with K48-linked diubiquitin (K48-Ub_2). In the UBA domain's structure alignment, I46/L65/L73 are equivalent to V15/L34/Y42 of p47. **(g)** Left, Western blot analysis of Ubxn7-depleted and undepleted extracts. Right, co-immunoprecipitates of FLAG-rUbxn7 variants from egg extracts in the presence or absence of p97i or Culi were analyzed with indicated antibodies. Lanes 1-21 and 22-42 are parts of the same experiment that were analyzed on separate Western blots processed and imaged in parallel under identical conditions. Note: neddylated Cul2 undergoes de-neddylation during FLAG immunoprecipitation procedure, and the degree of de-neddylation varies between different egg extracts preparations. We were not able to test whether Ubxn7 variants also recover Lrr1 from non-replicating extracts because a background band appeared at the position of Lrr1. Black arrowheads, neddylated Cul2; blue arrowheads, de-neddylated Cul2. IP, immunoprecipitation; N8-Cul2, neddylated Cul2. Other abbreviations as in Fig. 1a. Source data are provided as a Source Data file.

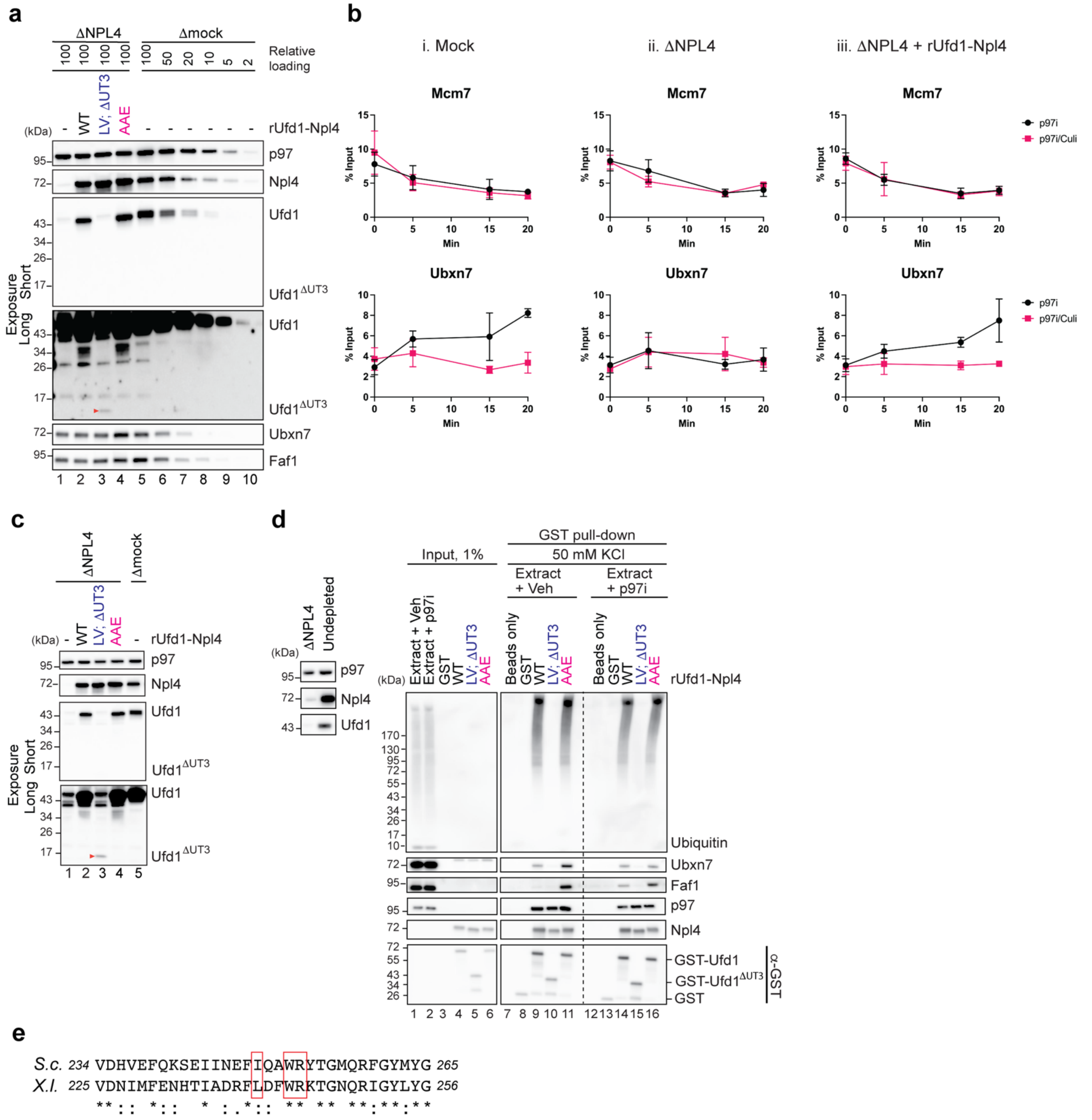

Supplementary Fig. 2: Ubxn7 binding to chromatin in Npl4-depleted extracts and characterization of Ufd1-Npl4 mutants. (a) Western blot analysis of mock and Ufd1-Npl4 depletions and recombinant Ufd1-Npl4 add-back. Related to Fig. 2a. For Ufd1 (long exposure), the contrast of the whole image was adjusted using ImageJ to enhance visibility of the bands. Red arrowhead indicates Ufd1$^{\Delta UT3}$. **(b)** Mcm7 and Ubxn7 ChIP from replication reaction in the presence of p97i or p97i/Culi in mock- or Npl4-deplted extracts. Extracts were supplemented with recombinant Ufd1-Npl4 (rUfd1-Npl4) as indicated. Data are presented as mean values of biologically independent replicates (n=4 for 0 and 5 min and n=3 for 15 and 20 min) $\pm$ s.d. **(c)** Western blot analysis of mock and Ufd1-Npl4 depletions and recombinant Ufd1-Npl4 add-back. Related to Fig. 2c. Red arrowhead indicates Ufd1$^{\Delta UT3}$. **(d)** Left, western blot analysis of Npl4 depletion. Right, co-immunoprecipitates of GST or GST-Ufd1 and Npl4 variants from non-replicating egg extracts in

the presence or absence of p97i were analyzed with indicated antibodies. The images are all part of the same Western blot, which was cropped to remove irrelevant information between lanes 6-7, Abbreviations as in Fig. 2d. **(e)** Alignment of a part of MPN domain of *Saccharomyces cerevisiae* (*S.c.*) and *Xenopus laevis* (*X.l.*) Npl4. “*”, identical residues; “:”, conserved substitutions; “.”, semi-conserved substitutions: red boxes, residues involved in ubiquitin unfolding in yeast and corresponding residues in *Xenopus* Npl4. Source data are provided as a Source Data file.

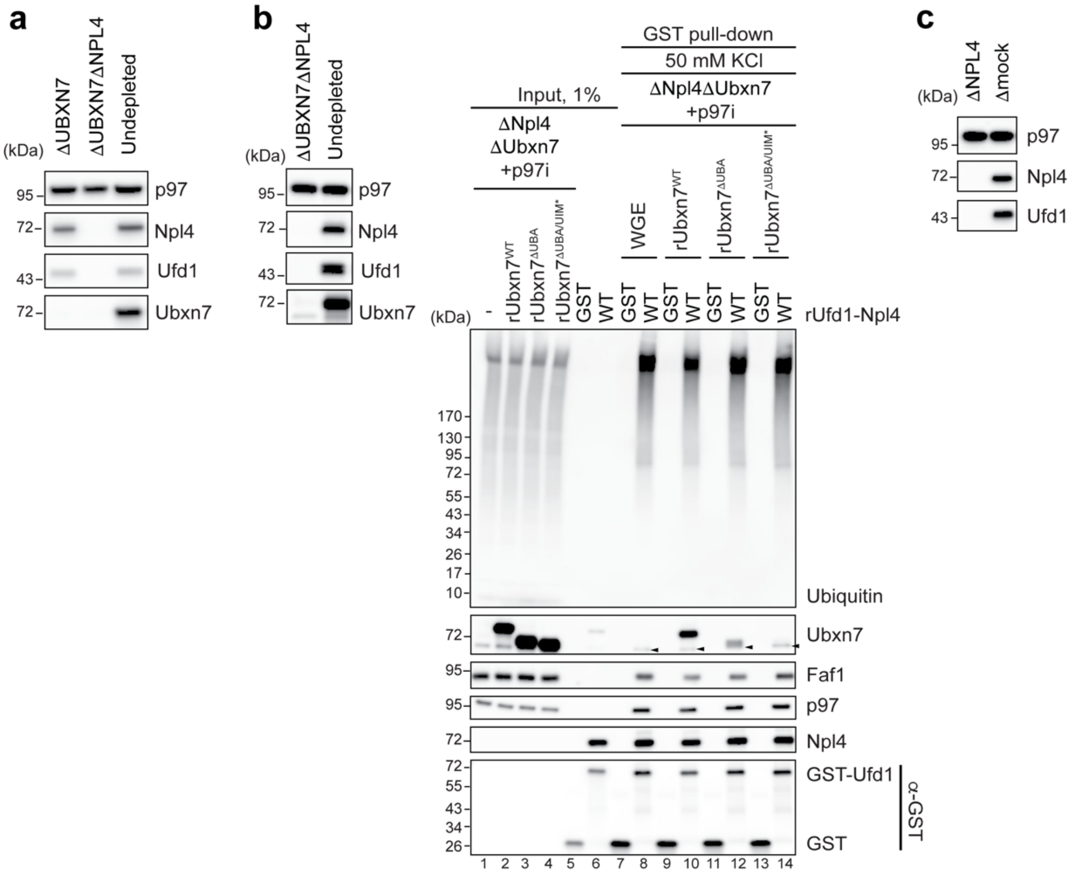

Supplementary Fig. 3: Analysis of complex formation between GST-Ufd1/Npl4 and Ubxn7 mutants. **(a)** Western blot analysis of Ubxn7-, Ubxn7/Npl4-depleted, and undepleted extracts. Related to Fig. 3b. **(b)** Left, Western blot analysis of Ubxn7/Npl4-depleted and undepleted extracts. Right, co-immunoprecipitates of GST or GST-Ufd1 and Npl4 heterodimer from non-replicating egg extracts supplemented with the indicated rUbxn7 variants expressed in wheat germ extract (WGE) in the presence of p97i were analyzed with the indicated antibodies. The images were cropped to remove irrelevant information. Black arrowheads, non-specific band. Abbreviations as in Fig. 2d. **(c)** Western blot analysis of mock- and Npl4-depleted extract. Related to Fig. 3c. Source data are provided as a Source Data file.

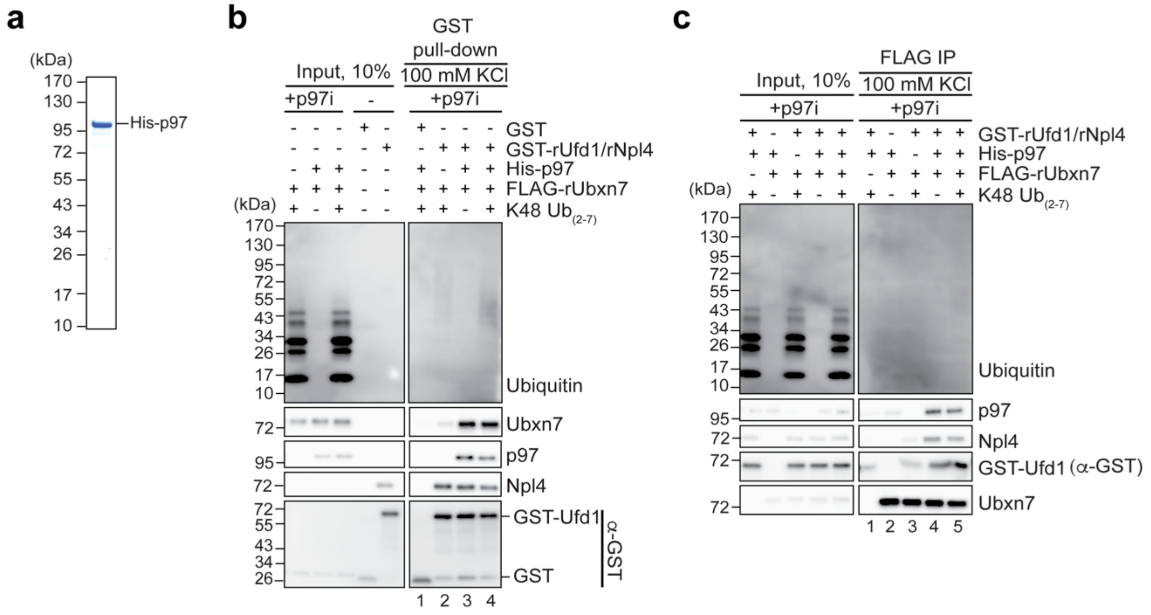

Supplementary Fig. 4: Reconstitution of the p97$^{Ufd1\text{-}Npl4\text{-}Ubxn7}$ complex *in vitro*. (a) Coomassie-stained SDS PAGE gel of recombinant His-p97. The contrast of the whole image was adjusted using Adobe Photoshop to enhance visibility of the band. **(b)** Co-immunoprecipitates of GST or GST-Ufd1 and Npl4 variants from a mixture of recombinant p97, Ubxn7, K48-linked ubiquitin chains (K48 Ub $_{(2\text{-}7)}$), and p97i were analyzed with the indicated antibodies. The images are all parts of the same Western blot, which was cropped to remove irrelevant information. **(c)** Co-immunoprecipitates of FLAG-Ubxn7 from a mixture of recombinant p97, GST-Ufd1/Npl4, K48-linked ubiquitin chains (K48 Ub $_{(2\text{-}7)}$), and p97i were analyzed with the indicated antibodies. The images are all parts of the same Western blot, which was cropped to remove irrelevant information. Source data are provided as a Source Data file.

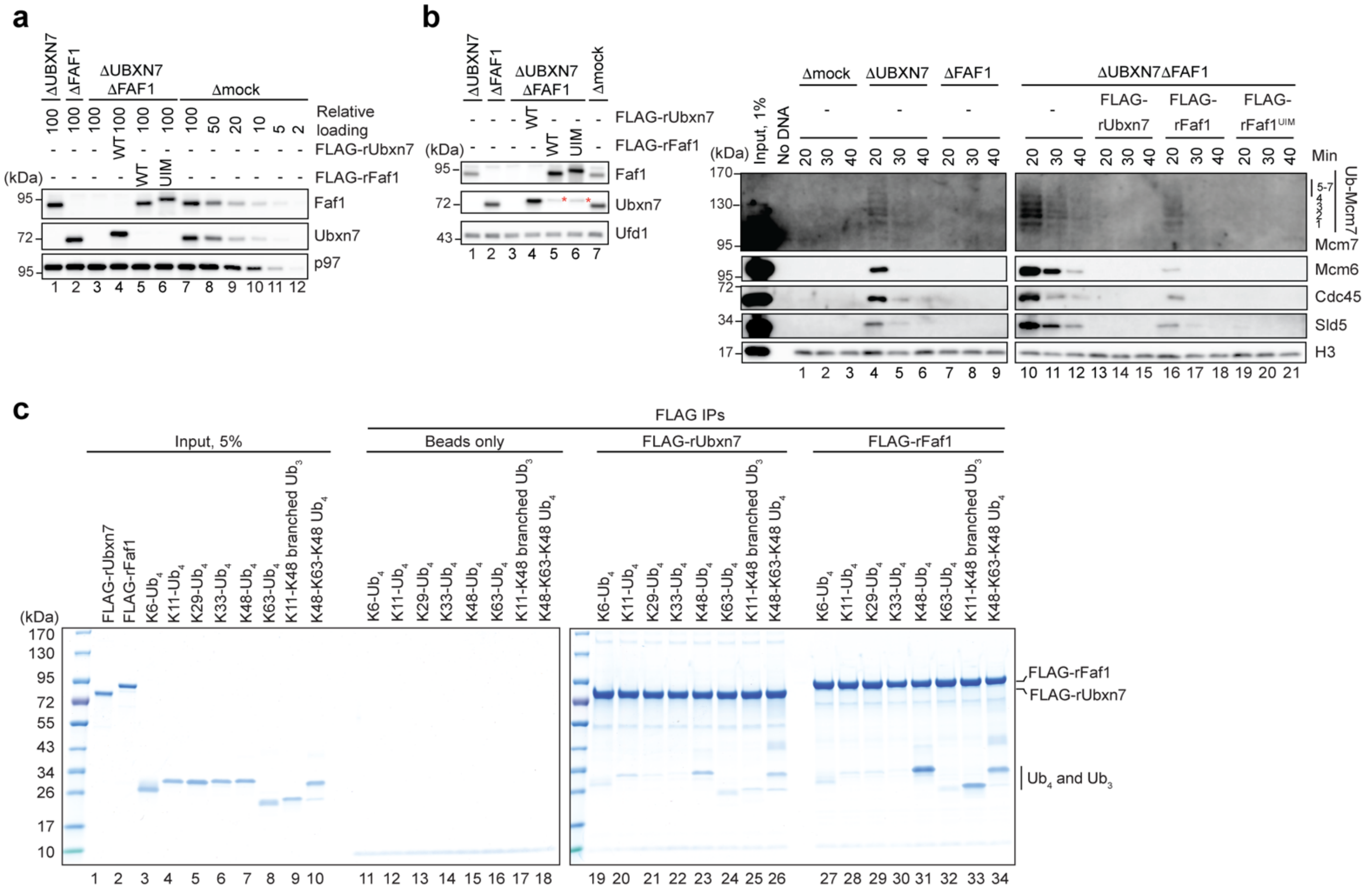

Supplementary Fig. 5: Comparison of Ubxn7 and Faf1 in CMG unloading and binding of ubiquitin chains. (a) Western blot analysis of mock-, Ubxn7-, Faf1-, and Ubxn7/Faf1-depleted extracts. Related to Fig. 4a. WT, wild-type Ubxn7 or Faf1, UIM, a Faf1^{UIM} chimera. **(b)** Left, same as Supplementary Fig. 4a. Red asterisks indicate cross-reaction of Ubxn7 antibodies with recombinant Faf1 proteins. For Faf1 and Ubxn7, the contrast of the whole image was adjusted using ImageJ to enhance visibility of the bands. Right, same as Fig. 4a, but plasmid DNA was recovered at 20, 30, and 40 min after replication initiation. Lanes 1-9 and 10-21 are parts of the same experiment that were analyzed on separate Western blots processed and imaged in parallel under identical conditions. For Mcm6, Mcm7, and Cdc45, the contrast of the whole image was adjusted using ImageJ to enhance visibility of the bands. **(c)** Analysis of co-immunoprecipitates of FLAG-rUbxn7 or FLAG-rFaf1 with the indicated ubiquitin chains. Ub_4, tetraubiquitin; Ub_3, triubiquitin. Proteins were detected by staining polyacrylamide gels with InstantBlue protein dye. Lanes 1-18 and 19-34 are parts of the same experiment that were analyzed on separate polyacrylamide gels and processed and imaged in parallel under identical conditions. The contrast of the whole image was adjusted using Adobe Photoshop to enhance visibility of the bands. Source data are provided as a Source Data file.

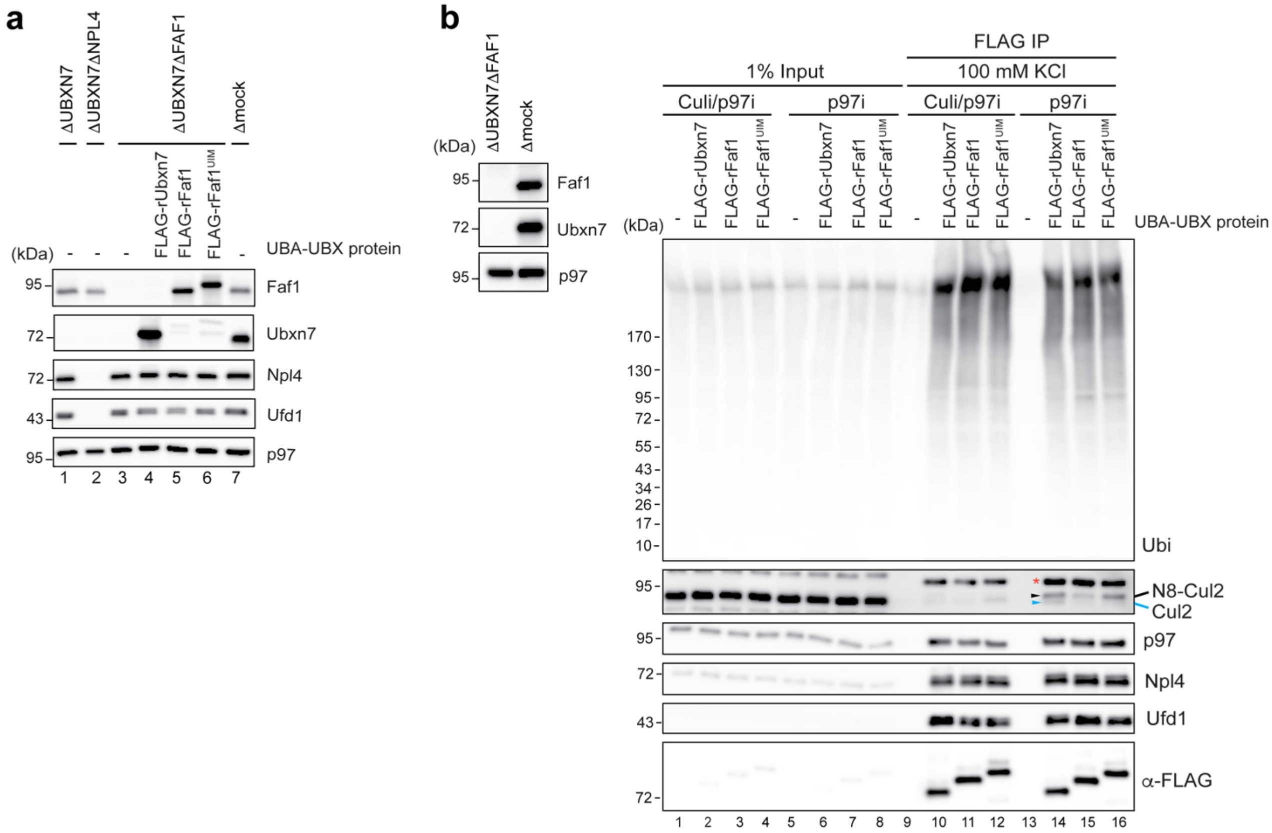

Supplementary Fig. 6: Comparison of Cul2-binding by Ubxn7 and Faf1 variants in egg extracts.
(a) Western blot analysis of mock-, Ubxn7-, Ubxn7/Npl4-, and Ubxn7/Faf1-depleted extracts supplemented with recombinant wild-type Ubxn7 or Faf1, and chimeric (Faf1^{UIM}) Faf1. Related to Fig. 4e. **(b)** Left, Western blot analysis of Ubxn7/Faf1-depleted and undepleted extracts. Right, co-immunoprecipitates of FLAG-Ubxn7, FLAG-Faf1^{WT}, and chimeric FLAG-Faf1^{UIM} from egg extracts in the presence or absence of Culi/p97i or p97i were analyzed with the indicated antibodies. Black arrowheads, neddylated Cul2; blue arrowheads, de-neddylated Cul2; red asterisk, cross-reacting band that is likely corresponds to p97. IP, immunoprecipitation; N8-Cul2, neddylated Cul2. Source data are provided as a Source Data file.

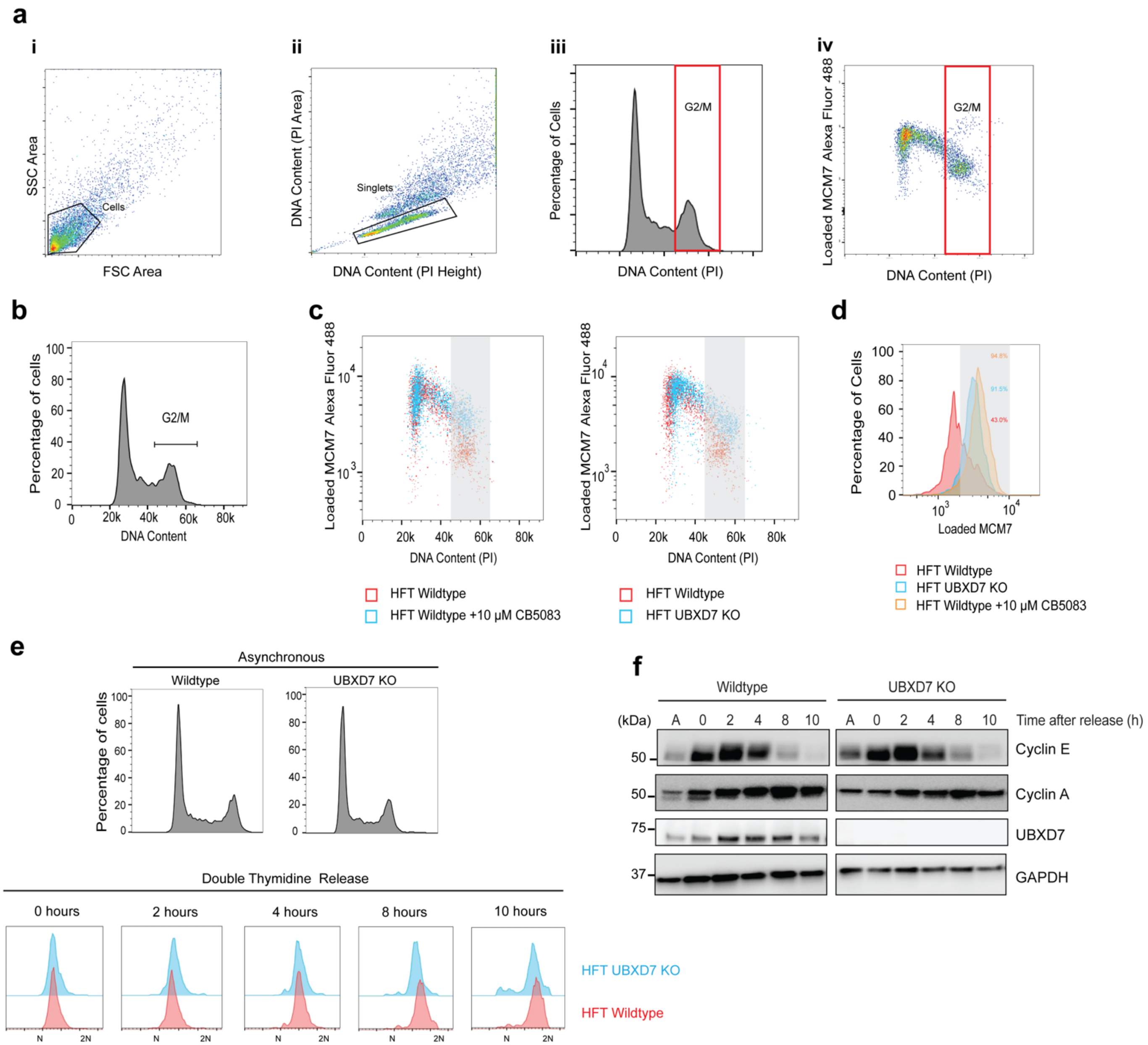

Supplementary Fig. 7: Analysis of MCM7 levels on chromatin and Cyclin A and E levels in wild-type and hUBXD7 KO HFT cells. (a) Example of flow cytometry gating with chromatin-extracted HFT WT cells. i, Gating to discriminate debris using FSC-area vs. SSC-area; ii, Singlet cells gating using PI-area vs. PI-height; iii, Cell cycle phases determined using histogram of PI signal; iv, G2/M-MCM loaded cells were identified by applying cell cycle gating to identify loaded MCM. **(b)** Distribution of DNA content. **(c)** Flow cytometry scatter plot showing chromatin-bound MCM7 (y axis) and DNA content (x axis). Shaded area indicates G2/M**.** (gate used for quantification: 3000 cells per condition) **(d)** Quantification of Supplementary Fig. 7c. Shaded area indicates G2/M cells with higher MCM intensity used for quantification in Fig. 5a. **(e)** Top, distribution of DNA content in asynchronous wild-type and hUBXD7 KO HFT cells. Bottom, distribution of DNA content in wild-type and hUBXD7 KO HFT cells, followed by double thymidine release at indicated time points. **(f)** Western blot analysis of Cyclin A and E levels in wild-type and hUBXD7 KO HFT cells, followed by double thymidine release at indicated time points. The images are all parts of the same Western blot, which was cropped to remove irrelevant information. A, asynchronous. Source data are provided as a Source Data file.

Supplementary Table 1

Oligonucleotides	Source
UBXD7 gRNA 5'-CACCGAATTAACCCCTTCAGCGCCG-3'	This study (ordered from IDT)
UBXD7 gRNA 5'-AAACCGGCGCTGAAGGGGTTAATTC-3'	This study (ordered from IDT)
ELO32_Ubxn7_fwd 5'-ATGTCTGGGGCTCCTTCG-3'	This study (ordered from IDT)
ELO33_Ubxn7_rev 5'-CTAATTCCTTTCCTGTACAAAGACAG-3'	This study (ordered from IDT)
OK8 5'-CTTGTCGTCATCGTCTTTGTAGTCCATG-3'	This study (ordered from IDT)
OK9 5'- GCGTTGCAGGAGACTCATTTTGATTCTGCAGTC-3'	This study (ordered from IDT)
OK10 5'-GGCACGGATAGCGGCTTCTAGTTG-3'	This study (ordered from IDT)
OK11 5'-GGCCGGCGCAAGCTTTCACACTTG-3'	This study (ordered from IDT)
OK12 5'-GAAGTTTGTGAGCAGCTCAAAGCGTTC-3'	This study (ordered from IDT)
OK15 5'-GCGGAGATGGCAGTCACCATGTTCGCGGATGGGGGTGGTATACCCGATGAGCCCAG-3'	This study (ordered from IDT)
OK16 5'-GTTGTGATTACATGCCTCCAGCATGTGCTTG-3'	This study (ordered from IDT)
OK18 5'-GCGACCGGTGCCACTGACAGTGTGGCCAAG-3'	This study (ordered from IDT)
OK19 5'-TGCTGTGAACTCCCTTAGGAGTTGCTGAGAGACTC-3'	This study (ordered from IDT)
OK21 5'-GGCATGGACTACAAAGACGATGACGACAAGGCCTCCAACACGGACAGAGAGATGATTCTG-3'	This study (ordered from IDT)
OK22 5'-GATCGTCAGTCAGTCACGATTTATTCCTTGGCTTCCAGAAATAGTGTTTCCTGAGG-3'	This study (ordered from IDT)
OK48 5'-TTTAACTTTAAGAAGGAGATATACCATGAGTGAATCTATCATTATCCGTATTCAG-3'	This study (ordered from IDT)
OK49 5'-GGTGTTTAAATGGTCTAGAAAGCTTTAGGTGTGAGGTAAGCGGCAC-3'	This study (ordered from IDT)
OK54 5'-CGGACCCTGAAACAGAACTTC-3'	This study (ordered from IDT)
OK55 5'-ATGAGTGAATCTATCATTATCCGTATTC-3'	This study (ordered from IDT)
OK58 5'-GGGGGTGGTATACCCGATGAG-3'	This study (ordered from IDT)
OK59 5'-GTTGTCTACATGACGATATTTTTGGCG-3'	This study (ordered from IDT)
OK60 5'-GGTTCGAAATTCGTTACGGTAGTAGC-3'	This study (ordered from IDT)
OK76 5'-CACAGCGAATACGCTGTTGACCTTGGG-3'	This study (ordered from IDT)
OK77 5'-CATCGGACCCTGAAACAGAACTTCCAGACC-3'	This study (ordered from IDT)
OK83 5' CTTCTATCCCCACCGCGCGATGCCATGGACTACAAAGACGATGACG-3'	This study (ordered from IDT)
OK84 5'-TCTTCACTGGTACCGAGCTCGAATTCTAATTCCTTTCCTGTACAAAGACAG-3'	This study (ordered from IDT)
OK85 5'-GAGGCCTTTTATGGGAAAGCAAGAG-3'	This study (ordered from IDT)
OK86 5'-CTGGTGACGTACTGGTATGCTTGTTA-3'	This study (ordered from IDT)
ChIP-F 5'-CGCTTTCTTCCCTTCCTTTCT-3'	This study (ordered from IDT)
ChIP-R 5'CTACGTGAACCATCACCCTAATC-3'	This study (ordered from IDT)
