# Supplementary material for: Cooperative assembly of p97 complexes involved in replication termination
Source: Nat Commun. 2022 Nov 3;13:6591. doi: 10.1038/s41467-022-34210-y (PMC9633789; doi:10.1038/s41467-022-34210-y)
Supplement: Supplementary file 3 — Reporting Summary [file 41467_2022_34210_MOESM3_ESM.pdf]

nature portfolio

Corresponding author(s): Johannes C Walter

Last updated by author(s): 10-06-2022

Reporting Summary

Nature Portfolio wishes to improve the reproducibility of the work that we publish. This form provides structure for consistency and transparency in reporting. For further information on Nature Portfolio policies, see our Editorial Policies and the Editorial Policy Checklist.

Statistics

For all statistical analyses, confirm that the following items are present in the figure legend, table legend, main text, or Methods section.

n/a	Confirmed	
☐	☒	The exact sample size (*n*) for each experimental group/condition, given as a discrete number and unit of measurement
☐	☒	A statement on whether measurements were taken from distinct samples or whether the same sample was measured repeatedly
☐	☒	The statistical test(s) used AND whether they are one- or two-sided<br>*Only common tests should be described solely by name; describe more complex techniques in the Methods section.*
☒	☐	A description of all covariates tested
☒	☐	A description of any assumptions or corrections, such as tests of normality and adjustment for multiple comparisons
☐	☒	A full description of the statistical parameters including central tendency (e.g. means) or other basic estimates (e.g. regression coefficient) AND variation (e.g. standard deviation) or associated estimates of uncertainty (e.g. confidence intervals)
☐	☒	For null hypothesis testing, the test statistic (e.g. *F*, *t*, *r*) with confidence intervals, effect sizes, degrees of freedom and *P* value noted<br>*Give P values as exact values whenever suitable.*
☒	☐	For Bayesian analysis, information on the choice of priors and Markov chain Monte Carlo settings
☒	☐	For hierarchical and complex designs, identification of the appropriate level for tests and full reporting of outcomes
☒	☐	Estimates of effect sizes (e.g. Cohen's *d*, Pearson's *r*), indicating how they were calculated

Our web collection on statistics for biologists contains articles on many of the points above.

Software and code

Policy information about availability of computer code

Data collection	Immunoblots were imaged using Amersham Imager 680 or a Bio-Rad Chemi-Doc Imaging system .<br>For flow cytometric analysis, samples were analyzed on a LSR II flow cytometer (BD Biosciences) equipped with 561 and 488-nm lasers.<br>qPCR for ChIP was performed on CFX384 Touch Real-Time PCR System.
Data analysis	Where indicated, the contrast of the whole image was adjusted using Adobe Photoshop 22.5.7 Release or ImageJ 1.52q.<br>Flow Cytometry data were analyzed using Flowjo version 10.8 (Tree Star)

For manuscripts utilizing custom algorithms or software that are central to the research but not yet described in published literature, software must be made available to editors and reviewers. We strongly encourage code deposition in a community repository (e.g. GitHub). See the Nature Portfolio guidelines for submitting code & software for further information.

Data

Policy information about availability of data

All manuscripts must include a data availability statement. This statement should provide the following information, where applicable:

- Accession codes, unique identifiers, or web links for publicly available datasets
- A description of any restrictions on data availability
- For clinical datasets or third party data, please ensure that the statement adheres to our policy

The authors declare that all data supporting the findings of this study are available within the article and its supplementary information files. Uncropped and unprocessed gel and immunoblot images are available as Source Data files.

Human research participants

Policy information about studies involving human research participants and Sex and Gender in Research.

Reporting on sex and gender	Not applicable
Population characteristics	Not applicable
Recruitment	Not applicable
Ethics oversight	Not applicable

Note that full information on the approval of the study protocol must also be provided in the manuscript.

Field-specific reporting

Please select the one below that is the best fit for your research. If you are not sure, read the appropriate sections before making your selection.

☒ Life sciences ☐ Behavioural & social sciences ☐ Ecological, evolutionary & environmental sciences

For a reference copy of the document with all sections, see nature.com/documents/nr-reporting-summary-flat.pdf

Life sciences study design

All studies must disclose on these points even when the disclosure is negative.

Sample size	For in vitro experiments: No sample size calculations were done. All in vitro experiments were repeated independently at least three times with the exception of experiments with negative results in Fig. 4c, Supplementary Fig. 1e, Supplementary Fig. 4c, which were performed twice.<br><br>Mammalian cell studies: No sample size calculations were done. For FACS, 3000 single cells were analyzed for each of the three independent experiments. For chromatin fractionation and Cyclin levels analysis, 25 µg total protein was resolved by SDS-PAGE in each of the three independent experiments. All mammalian studies were performed three times with a representative result shown.
Data exclusions	No data were excluded from the analysis.
Replication	To verify reproducibility all experiments were performed at least three times, with the exception of experiments in Fig. 4c, Supplementary Fig. 1e, Supplementary Fig. 4c, which produced negative results and were performed twice. All attempts of replication were successful.
Randomization	This study did not involve allocations into experimental groups and randomization was not relevant.
Blinding	This study did not involve allocations into experimental groups and blinding was not relevant.

Reporting for specific materials, systems and methods

We require information from authors about some types of materials, experimental systems and methods used in many studies. Here, indicate whether each material, system or method listed is relevant to your study. If you are not sure if a list item applies to your research, read the appropriate section before selecting a response.

Materials & experimental systems

n/a	Involved in the study
☐	☒ Antibodies
☐	☒ Eukaryotic cell lines
☒	☐ Palaeontology and archaeology
☐	☒ Animals and other organisms
☒	☐ Clinical data
☒	☐ Dual use research of concern

Methods

n/a	Involved in the study
☒	☐ ChIP-seq
☐	☒ Flow cytometry
☒	☐ MRI-based neuroimaging

Antibodies

Antibodies used

All antibodies are available upon request from the authors or from commercial suppliers.
Got Anti-Rabbit Peroxidase-conjugate Secondary Antibodies (IgG,H+L), Jackson ImmunoReserach cat no. 111-035-003
Mouse Anti-Rabbit Peroxidase -conjugate Secondary Antibody (IgG, Light Chain Specific) Jackson ImmunoResearch cat. no. 211-032-171
Rabbit anti-mouse horseradish peroxidase-conjugated antibodies (Jackson ImmunoResearch, 315-035-003)
Goat anti-rabbit secondary antibodies (Promega, W4011)
Goat anti-mouse secondary antibodies (Promega, W4021)
Goat anti-Rabbit IgG (H+L) Cross-Adsorbed Secondary Antibody, Alexa Fluor™ 488 (Life Technology, A-11008)
hCDC45L Novus Biologicals NBP2-67897, Clone JJ091-04
hMCM7 (Western blot) Cell Signaling Technology Cat# 4018S
hUBXD7 Thermo Scientific Cat# PA5-61972
hMCM7 (Flow Cytometry) Bethyl Laboratories Cat# A302-584A-M
hMCM3 (Cell Signaling Technology, 4012S)
GST Cell Signaling Cat#2624, clone 26H1
Ubiquitin Santa Cruz Biotechnology Cat# sc-8017, clone P4D1
Ubiquitin (used in Figure 6B) EMD Millipore Cat# 04-263, clone FK2
GAPDH Santa Cruz Biotechnology Cat# sc-47724, clone 0411
H3 EMD Millipore Cat# 06-755
FLAG (Wu et al., 2019), (Walter laboratory, Harvard Medical School, USA)
Mcm6 (Dewar et al., 2017), (Walter laboratory, Harvard Medical School, USA)
Sld5 (Dewar et al., 2017), (Walter laboratory, Harvard Medical School, USA)
Lrr1 (Dewar et al., 2017), (Walter laboratory, Harvard Medical School, USA)
Cul2 (Dewar et al., 2017), (Walter laboratory, Harvard Medical School, USA)
Mcm7 (Walter and Newport, 2000), (Walter laboratory, Harvard Medical School, USA)
Cdc45 (Mimura and Takisawa, 1998), (Walter laboratory, Harvard Medical School, USA)
Faf1 (Western blot) Abcam 202298
H3 Cell Signaling 9715S
Ubxn7 This study, Walter laboratory, Harvard Medical School, USA
Npl4 (Immunodepletion) This study , Walter laboratory, Harvard Medical School, USA
Faf1 (Immunodepletion) This study, Walter laboratory, Harvard Medical School, USA
p97 (Heubes and Stemmann, 2007), (Olaf Stemmann, University of Bayreuth, Germany)
Ufd1 (Heubes and Stemmann, 2007), (Olaf Stemmann, University of Bayreuth, Germany)
Npl4 (Western blot) (Heubes and Stemmann, 2007), (Olaf Stemmann, University of Bayreuth, Germany)

Validation

All custom antibodies against Xenopus proteins (Mcm6, Sld5, Lrr1, Cul2, Mcm7, Cdc45, Faf1, Ubxn7, Npl4, p97, Ufd1) were validated by Western blotting of the respective antigen and the detection of the band of the expected size by Western blotting Xenopus egg extracts .
Custom antibodies against FLAG peptide were validated by western blotting FLAG-tagged recombinant protein (Wu et al., 2019).
Goat Anti-Rabbit Peroxidase-conjugate Secondary antibodies were validated by manufacturer in antigen-binding assay, Western blotting, and/or Elisa.
Rabbit anti-mouse horseradish peroxidase-conjugated antibodies (Jackson ImmunoResearch, 315-035-003) were validated by manufacturer. Based on immunoelectrophoresis and/or ELISA, the antibody reacts with whole molecule mouse IgG. It also reacts with the light chains of other mouse immunoglobulins. No antibody was detected against non-immunoglobulin serum proteins. The antibody may cross-react with immunoglobulins from other species.
Mouse Anti-Rabbit Peroxidase-conjugate Secondary antibodies were validated by manufacturer in antigen-binding assay, Western blotting, and/or Elisa.
Goat anti-rabbit secondary antibodies (Promega, W4011) were validated to bind to both heavy and light chains for all rabbit IgG subclasses by manufacturer. Recommended applications - WB, ELISA, dot blotting.
Goat anti-mouse IgG (H+L), HRP Conjugate (Promega, W4021) antibodies were validated to bind to both heavy and light chains for all mouse IgG subclasses by manufacturer. Recommended applications - WB, ELISA, dot blotting.
Goat anti-Rabbit IgG (H+L) Cross-Adsorbed Secondary Antibody, Alexa Fluor™ 488 (Life Technology, A-11008) is validated for ICC/IF/IHC by manufacturer.
hCDC45L Novus Biologicals NBP2-67897, Clone JJ091-04 is validated for Western Blot, Flow Cytometry, Immunohistochemistry, Immunocytochemistry/Immunofluorescence, Immunohistochemistry-Paraffin by manufacturer.
hMCM7 (Western blot) Cell Signaling Technology Cat# 4018S is validated for Western blotting by manufacturer.
hUBXD7 Thermo Scientific Cat# PA5-61972 is validated by manufacturer for ICC/IF, IHC, WB, and by relative expression.
hMCM7 (Flow Cytometry) Bethyl Laboratories Cat# A302-584A-M is validated by WB
hMCM3 (Cell Signaling Technology, 4012S) is validated for WB, IP, IF/IC by manufacturer

GST Cell Signaling Cat# 26H1 is validated by manufacturer by WB, IP, and IF.
Ubiquitin Santa Cruz Biotechnology Cat# sc-8017 P4D1 is validated by WB. IHC, IF by manufacturer.
Ubiquitin (used in Figure 6B) EMD Millipore Cat# 04-263, clone FK2 is validated for ELISA, IP, WB, IF by manufacturer.
GAPDH Antibody Cat# sc-47724, clone 0411 is validated for detection of GAPDH of human origin by WB, IP, IF and IHC(P) by manufacturer.
H3 EMD Millipore Cat# 06-755 is validated in ChIP, ICC & WB by manufacturer.
Cyclin A (H-432) Santa Cruz Cat# SC-751 is validated for detection of cyclin A and cyclin A1 of mouse, rat and human origin by WB, IP, IF and ELISA by manufacturer (as of 9/2022, discontinued by manufacturer).
Cyclin E (C-19) Santa Cruz Cat# SC-198 is validated for recommended for Western Blotting , IP, IF, IHC, and ELISA by manufacturer.
Faf1 (Western blot) Abcam Cat# 202298 is validated in Flow Cyt (Intra), IHC-P, WB, ICC/IF, IP by manufacturer.
H3 Cell Signaling Cat# 9715S is validated for WB, IP, ChIP, IP, IF by manufacturer.

Eukaryotic cell lines

Policy information about cell lines and Sex and Gender in Research

Cell line source(s): HeLa T-Rex cell line (Thermo Fisher cat#R71407) with introduced Flp-in site (Flp-In™ T-REx™ Core Kit, ThermoFisher, Catalog number: K650001) is a gift from Brian Raught, University of Toronto, Canada.

Authentication: None of the cell lines used were authenticated.

Mycoplasma contamination: Cells were routinely tested for mycoplasma infection using MycoAlert. All cell lines tested negative for mycoplasma contamination.

Commonly misidentified lines (See ICLAC register): No commonly misidentified cell lines were used in the study.

Animals and other research organisms

Policy information about studies involving animals; ARRIVE guidelines recommended for reporting animal research, and Sex and Gender in Research

Laboratory animals: Xenopus laevis were purchased from Nasco. Females used for egg collection were aged >2 years. Males used for sperm chromatin isolation were aged >1 year.

Wild animals: This study did not involve wild animals.

Reporting on sex: This study did not involve sex-based analysis.

Field-collected samples: This study did not involve field-collected samples.

Ethics oversight: Animal work performed at Harvard Medical School was approved by the Harvard Medical Area Standing Committee on Animals (HMA IACUC Study ID I IS00000051-6, approved 10/23/2020). The institution has an approved Animal Welfare Assurance (#D16-00270) from the NIH Office of Laboratory Animal Welfare.

Note that full information on the approval of the study protocol must also be provided in the manuscript.

Flow Cytometry

Plots

Confirm that:

☒ The axis labels state the marker and fluorochrome used (e.g. CD4-FITC).

☒ The axis scales are clearly visible. Include numbers along axes only for bottom left plot of group (a 'group' is an analysis of identical markers).

☒ All plots are contour plots with outliers or pseudocolor plots.

☒ A numerical value for number of cells or percentage (with statistics) is provided.

Methodology

Sample preparation: Chromatin extraction and fixation of HeLa Flp-in T-REX cells for flow cytometry were performed as described previously (Matson et al., 2017). Briefly, cells were trypsinized and resuspended into DMEM containing 10% FBS as single cells. Cells were centrifuged at 2000 xg for 5 min and resuspended in PBS and centrifuged again at 2000 xg for 3 min. Cells were lysed in 500ml of ice-cold CSK buffer (300 mM Sucrose, 300 mM NaCl, 3 mM MgCl2, 1 mM PIPES pH 7.0) containing 0.5 % Triton X-100, HALT protease inhibitors (Thermo Scientific) and phosphatase inhibitor (1mM sodium orthovanadate) for 7 min on ice. 1 ml of 1% BSA-PBS was added to the samples, mixed, and cells were centrifuged at 2000 xg for 3 min. Cells were fixed by resuspending thoroughly in 4% paraformaldehyde (Electron Microscopy Sciences) in PBS for 15 min at room temperature in the dark. 1ml of 1% BSA-PBS was added, mixed by gentle pipetting and cells were collected at 2000 xg for 7 min. Samples were resuspended in primary antibody (1:200 µl for MCM7) diluted in 1% BSA-PBS containing 0.1% NP-40 and rocked overnight at 4C in the dark. 1 ml of 1% BSA-PBS containing 0.1% NP-40 was added, cells were mixed gently and collected at

2000 xg for 7 min. Cells were resuspended in 300µl of secondary antibody (Life Technology, Alexa Fluor 488, 1:1000) diluted in 1% BSA-PBS containing 0.1% NP-40. Cells were incubated by rocking at room temperature for 1 hr in the dark. 1ml of 1% BSA-PBS was added, cells were mixed gently, and cells were collected at 2000 xg for 7 min. Finally, cells were resuspended in 1% BSA-PBS containing 0.1% NP-40 with 5 µg/ml of Propidium Iodide (PI) (Sigma) and 200 µg/ml RNAse A (Sigma) for 30 min at room temperature in the dark to label DNA.

Instrument

For flow cytometric analysis, samples were analyzed on a LSR II flow cytometer (BD Biosciences) equipped with 561 and 488-nm lasers.

Software

Data was analyzed using Flowjo version 10.8 (Tree Star).

Cell population abundance

3000 singlet, live cells were analyzed after gating for each of the three independent experiments. Specificity of MCM7 labeling in the gated population was determined by sorting cells stained with only the secondary antibody to ensure purity and specificity of the MCM signal.

Gating strategy

Live cells were gated using forward and side scatter. Single cells were gated using PI area vs PI height. 3000 single cells were analyzed for each of the three independent experiments.

☒ Tick this box to confirm that a figure exemplifying the gating strategy is provided in the Supplementary Information.
